# Editorial: Applications of Metagenomics in Studying Human Cancer

**DOI:** 10.3389/fgene.2021.760141

**Published:** 2021-09-16

**Authors:** Guohua Huang, Jialiang Yang, Lei Chen, Taoyang Wu

**Affiliations:** ^1^ School of Electrical Engineering, Shaoyang University, Shaoyang, China; ^2^ Geneis (Beijing) Co., Ltd., Beijing, China; ^3^ College of Information Engineering, Shanghai Maritime University, Shanghai, China; ^4^ School of Computing Sciences, University of East Anglia, Norwich, United Kingdom

**Keywords:** metagenomics, cancer, microbe, next generation sequencing, functional gene screening

Metagenomics is defined as analysis of DNA obtained directly from the environment (Hugenholtz and Tyson, 2008) and is also referred to as environmental genomics, ecogenomics or community genomics (Handelsman 2004). Recent studies have reported that microbial infection might be responsible for about 16.1% of cancers (Banerjee et al., 2015). For example, Chronic infections with hepatitis B virus (HBV) and hepatitis C virus (HCV) might be closely associated with cirrhosis, liver cancers and Cholangiocarcinoma (Perz et al., 2006; Matsumoto et al., 2014), HCV infection contributed much to the development of hepatocellular carcinoma (Saito et al., 1990), and a large portion of human papilloma virus (HPV) infection played a crucial role in the development of cervical carcinoma (An et al., 2003). More importantly, roles of the microbiota differ significantly with types of cancers. Therefore, to explore metagenomics of cancers is helpful to shed light on the underlying mechanisms of cancer as well as promote microbe-mediated cancer drug development.

With development of sequencing techniques, many methods have been developed for analyzing metagenomics of cancers in the past decade, including experimental and computational methods (Zhang et al., 2019; Fadiji and Babalola, 2020). However, there are still barriers to be solved in the exploring metagenomics-related cancers (Laudadio et al., 2019). Both computational and experimental methods on metagenomics are still in their early stages and more focus should be put into this promising area. This Research Topic serves as a forum to discuss new methods of analyzing metagenomics-related cancers.

Endometrial cancer (EC) is one of the most common female malignant tumors (Ryan et al., 2005; Liu et al., 2019). The presence of poor prognostic factors driving tumor recurrence contributed much to mortality of EC patients (Coll-De La Rubia et al., 2020). Zhang et al. employed the bioinformatics tools to explore role of E2F family in EC. Zhang et al. found that expressions of E2F1, E2F2, E2F3, E2F7, and E2F8 were significantly upregulated and the expressions of E2F4 were downregulated in EC tissues. This finding implied clinical potential of E2F family in preventing and treating EC.

Cancer of unknown primary site (CUP) is a well-recognized and heterogeneous clinical disorder, one of the 10 most common malignancies in developed countries, accounting for 3–5% of cancers in both men and women and (Pavlidis and Pentheroudakis, 2010). Identifying tissue of origin is crucial to early diagnose and treat CUP (Zhao et al., 2020). Zhang et al. developed an XGBoost-based method for predicting cancer tissue-of-origin by using copy number variations. This method might be potential for identifying the actual clinic pathological status of specific cancer.

Breast cancer (BC) is the most common female malignant tumor, which is of extremely high morbidity and mortality (Wang et al., 2017). The cancer cells interact inevitably with the immune system during the process of cancer development, which might inhibit or enhance tumor growth (Vinay et al., 2015). Understanding deeply immune evasion mechanism of BC is critical to develop immunotherapies for breast cancer. Chen et al.presented a multi-source data fusion scheme to investigate regulatory mechanism of BC immune evasion.

Long non-coding RNA is a type of RNA with more than 200 nucleotides, which are not translated into proteins (Perkel 2013). Accumulating evidences showed that lncRNAs were closely associated with human cancers (Gutschner and Diederichs, 2012). The aberrance of lncRNA was involved in a large variety of cancers (Gibb et al., 2011). Little was known about functional roles of the lncRNAs in the cancers. Zhu et al. developed a decision tree-based method for identifying cancer-related lncRNAs. Zhu et al. also used the GO and the KEGG pathway to encode lncRNA, and employed Minimum Redundancy Maximum Relevance (mRMR) (Peng et al., 2005) to select discriminative GO and KEGG terms. The selected GO and KEGG terms were helpful to shed light on regulating mechanisms of lncRNAs in tumorigenesis. The glioma is a type of brain tumor, accounting for 30% of all brain tumors, and 80% of malignant brain tumours (Goodenberger and Jenkins, 2012). Xu et al. explored the prognostic value of MEG3 which is a maternally expressed and imprinted long non-coding RNA gene, and investigated correlations with immune infiltrates in Gliomas. Xu et al. found that expressions of MEG3 were strongly related to the multiple immune markers in gliomas, especially in low-grade glioma, implying potential immunotherapeutic target for gliomas.

Clear cell renal cell carcinoma (ccRCC) is the most frequent subtype of kidney cancer (Network 2013). Most of ccRCC are of metastase which is main cause of mortality for ccRCC (Casuscelli et al., 2019). Finding difference in expressed profiles of genes in primary metastatic ccRCC is fundamental to understand metastasis mechanism of ccRCC. Gao et al. identified a metastasis-associated gene signature of ccRCC by bioinformatics analysis on two gene expression profiles (GSE105261 and GSE85258) which were downloaded from the GEO (Gene Expression Omnibus) database (https://www.ncbi.nlm.nih.gov/geo/). This might provide new insights into therapeutic and early diagnostic biomarkers of ccRCC.

Hepatocellular Carcinoma (HCC) is the most common primary liver cancer, with which over 80% patients occurred in the developing countries (Yang and Roberts, 2010). Autophagy is an evolutionarily conserved intracellular mechanism (Yazdani et al., 2019), playing versatile roles in cellular process including removal of unnecessary or dysfunctional components (Klionsky 2008) and recycling of cellular components (Mizushima and Komatsu, 2011; Kobayashi 2015). It was reported that autophagy played dual roles in the HCC. The autophagy can inhibit but can promote development of HCC. HCC is of poor prognosis. Wang et al. presented a multivariate Cox model for exploring the influence of autophagy-related genes (AAGs) on the prognosis of HCC. This model is potential to promote the overall survival rate of HCC patients.

Alzheimer’s disease (AD) is a neurodegenerative disease and its mechanism is poorly understood (Burns and Iliffe, 2009). Single nucleotide polymorphism (SNP) refers to the substitution of a single nucleotide at a specific position in the genome. SNP was associated with many disease including AD. Zhou et al. presented a multiple-step method for exploring association between SNP and brain region of interest related to AD.
